# α-Bisabolol, a Dietary Bioactive Phytochemical Attenuates Dopaminergic Neurodegeneration through Modulation of Oxidative Stress, Neuroinflammation and Apoptosis in Rotenone-Induced Rat Model of Parkinson’s Disease

**DOI:** 10.3390/biom10101421

**Published:** 2020-10-08

**Authors:** Hayate Javed, M. F. Nagoor Meeran, Sheikh Azimullah, Lujain Bader Eddin, Vivek Dhar Dwivedi, Niraj Kumar Jha, Shreesh Ojha

**Affiliations:** 1 Department of Anatomy, College of Medicine and Health Sciences, United Arab Emirates University, Al Ain, P.O. Box 17666, UAE; h.javed@uaeu.ac.ae; 2 Department of Pharmacology and Therapeutics, College of Medicine and Health Sciences, United Arab Emirates University, Al Ain, P.O. Box 17666, UAE; nagoormeeran1985@uaeu.ac.ae (M.F.N.M.); azim.sheikh@uaeu.ac.ae (S.A.); 201970113@uaeu.ac.ae (L.B.E.); 3 Center for Bioinformatics, Computational and Systems Biology, Pathfinder Research and Training Foundation, 30/7 and 8, Knowledge Park III, Greater Noida, Uttar Pradesh 201308, India; vivek_bioinformatics@yahoo.com; 4 Department of Biotechnology, School of Engineering & Technology (SET), Sharda University, Plot No.32-34, Knowledge Park III, Greater Noida, Uttar Pradesh 201310, India; niraj.jha@sharda.ac.in; 5 Zayed Center for Health Sciences, United Arab Emirates University, Al Ain, P.O. Box 17666, UAE

**Keywords:** rotenone, Parkinson’s disease, α-bisabolol, oxidative stress, apoptosis, inflammation

## Abstract

Rotenone (ROT), a plant-derived pesticide is a well-known environmental neurotoxin associated with causation of Parkinson’s disease (PD). ROT impairs mitochondrial dysfunction being mitochondrial complex-I (MC-1) inhibitor and perturbs antioxidant-oxidant balance that contributes to the onset and development of neuroinflammation and neurodegeneration in PD. Due to the scarcity of agents to prevent the disease or to cure or halt the progression of symptoms of PD, the focus is on exploring agents from naturally occurring dietary phytochemicals. Among numerous phytochemicals, α-Bisabolol (BSB), natural monocyclic sesquiterpene alcohol found in many ornamental flowers and edible plants garnered attention due to its potent pharmacological properties and therapeutic potential. Therefore, the present study investigated the neuroprotective effects of BSB in a rat model of ROT-induced dopaminergic neurodegeneration, a pathogenic feature of PD and underlying mechanism targeting oxidative stress, inflammation and apoptosis. BSB treatment significantly prevented ROT-induced loss of dopaminergic neurons and fibers in the substantia nigra and striatum respectively. BSB treatment also attenuated ROT-induced oxidative stress evidenced by inhibition of MDA formation and GSH depletion as well as improvement in antioxidant enzymes, SOD and catalase. BSB treatment also attenuated ROT-induced activation of the glial cells as well as the induction and release of proinflammatory cytokines (IL-1β, IL-6 and TNF-α) and inflammatory mediators (iNOS and COX-2) in the striatum. In addition to countering oxidative stress and inflammation, BSB also attenuated apoptosis of dopaminergic neurons by attenuating downregulation of anti-apoptotic protein Bcl-2 and upregulation of pro-apoptotic proteins Bax, cleaved caspases-3 and 9. Further, BSB was observed to attenuate mitochondrial dysfunction by inhibiting mitochondrial lipid peroxidation, cytochrome-C release and reinstates the levels/activity of ATP and MC-I. The findings of the study demonstrate that BSB treatment salvaged dopaminergic neurons, attenuated microglia and astrocyte activation, induction of inflammatory mediators, proinflammatory cytokines and reduced the expression of pro-apoptotic markers. The in vitro study on ABTS radical revealed the antioxidant potential of BSB. The results of the present study are clearly suggestive of the neuroprotective effects of BSB through antioxidant, anti-inflammatory and anti-apoptotic properties in ROT-induced model of PD.

## 1. Introduction

Parkinson’s disease (PD) is one of the most prevalent slow-progressing, chronic age-related neurodegenerative disease characterized by degeneration of dopaminergic (DA) neurons in the substantia nigra (SN) [1]. The clinical motor symptoms such as muscular rigidity, resting tremors, bradykinesia and postural inability appears usually after degeneration of more than 80% of the DA neurons [2]. The exact pathogenic mechanisms of PD remain elusive, but over the years it has been convincingly recognized that occurrence of oxidative stress plays a vital role in the initiation and progression of dopaminergic degeneration [3]. In PD brain, oxidative stress is greatly increased [4] because of high lipid contents in the brain tissues. Over production of reactive oxygen species (ROS) and nitric oxide (NO) prompts cellular damage through lipid peroxidation, protein oxidation, mitochondrial dysfunction and neuroinflammation following glial cells activation [5]. All these biochemical events consequently lead to dopaminergic neurodegeneration and appearance of motor symptoms of PD. Hence, oxidative stress, inflammation and apoptosis represent an important therapeutic target for pharmacological manipulation in PD [6].

There is no cure for PD except symptomatic treatment using levodopa as primary drug for PD but not devoid of significant side effects [7]. Considering the inadequacy of therapeutic agents, neuroprotection at early stage is a crucial strategy to prevent or delay the progression of PD. In recent years, many of the naturally occurring plant-derived chemicals termed as phytochemicals found to mitigate oxidative stress, inflammation and apoptosis. In past few years, phytochemicals generated interest for therapeutic and preventive strategy in PD and can be a useful novel nutraceutical approach in neuroprotection [8]. Growing evidences have shown that dietary phytochemicals also popular as nutraceuticals can attenuate neurodegenerative diseases through numerous mechanisms [9]. Many phytochemicals such as berberine exhibits neuroprotective effects by activation of the PI3K/Akt/Nrf2 pathway by scavenging free radicals. Moreover, it has been observed that berberine exhibits anti-apoptotic effects by reducing the expression of caspase 1 and 3, Bax as well as upregulation of Bcl-2 [10]. Curcumin has various medicinal properties and therefore it is used in the treatment of neurodegenerative diseases including PD [11]. One of the mechanisms that have been suggested for curcumin is that it has ability to bind to amyloid plaques by inhibiting NF-κβ thus reducing the pathogenesis of AD [12]. It has also been shown that curcumin helps in regenerating neurons by activating Trk/PI3K signaling pathways, which elevate BDNF levels in a PD model [13]. Quercetin, a phenolic compound showed to protect from neurodegenerative disease by regulating NF-κB which play important role in ameliorating inflammatory processes involved in neurodegenerative disease [14]. Quercetin has shown to enhance the biogenesis of mitochondria and this is important because mitochondrial dysfunction leads to neuronal degeneration by depletion of cellular ATP levels and ROS generation [15].

Among numerous bioactive dietary phytochemicals, α-Bisabolol (BSB), also referred to as levomenol generated interest due to its therapeutic potential and multiple pharmacological properties such as anti-inflammatory [16], antioxidant [17], anti-infective [18], antitumor [19], cardioprotective [20] and anti-nociceptive [16]. BSB is unsaturated sesquiterpene alcohol with abundant presence in sage (*Salvia runcinata*), anyme wood oil (*Myoporum crassifolium*), chamomile (*Matricaria chamomilla*), candeia (*Ereman thusery-thropappus*), and negramina (*Siparuna guianensis*) [21]. For a long time, BSB is used mostly in dermal preparations due to its potent skin and wound healing properties [22]. BSB has been considered a relatively safe compound for human consumption and well used in foods and beverages as additive, preservative and for aroma [22]. Moreover, BSB has been well documented for its potent pharmacological properties and therapeutic potential in treating human diseases.

BSB has shown protective effects against cellular model of Alzheimer’s diseases in Aβ induced neurotoxicity in PC12 cells [23]. Recently, the neuroprotective effects of BSB supplementation has been demonstrated against rotenone (ROT)-induced neurotoxicity in Drosophila melanogaster (fruit fly) by attenuating mitochondrial dysfunction along with improving antioxidant defense [24]. In purview of the reported neuroprotective potential, the present study was carried out to evaluate the therapeutic potential of BSB in an experimental model in rats injected ROT systemically for long time to induce dopaminergic neurodegeneration mimicking the pathological hallmarks of PD [25]. The ROT model garnered attention due to its reproducibility, feasibility and its nature of being an environmental toxin, which is considered a major culprit of neurodegeneration. ROT-induced dopaminergic neurodegeneration is a well-established and widely accepted experimental model of PD to investigate and evaluate the neuroprotective potential and mechanism of natural compounds [26]. The present study was aimed to investigate the neuroprotective role of BSB in the ROT-induced rat model of PD, a physiologically relevant animal model to elucidate the effects of BSB on oxidative stress, inflammation and apoptosis in a comprehensive manner.

ROT isolated from the roots or leaves of plants within the *Lonchocarpus* and *Derris* genera is a naturally occurring pesticide and insecticides commonly used against insects and nuisance fish in lakes worldwide [27]. Due to its high lipophilicity, it easily crosses all membranes including the blood-brain barrier to reach into the brain cells independent of any transporters (unlike MPP^+^) [28]. ROT-induced degeneration in rodents reproduces degeneration as seen in clinical PD, with dopaminergic damage of nerve terminals in the striatum and cell bodies in SN [26]. In addition to that, ROT causes mitochondrial complex-I (MC-I) inhibition that leads to increased oxidative stress, loss of energy, inflammation, sustained activation of glial cells and nigrostriatal degeneration [29]. The findings of the present study demonstrated the neuroprotective effects of BSB in ROT-induced PD in rats mediating antioxidant, anti-inflammatory and anti-apoptotic properties.

## 2. Materials and Methods

### 2.1. Drugs and Chemicals

The antibodies used in the present study are provided in Table 1. Secondary antibodies including biotinylated donkey anti-rabbit (Jackson Immunoresearch, West Grove, PA, USA) (catalog number: 711-065-152), and Alexa fluor 488-conjugated goat anti-rabbit (Life Technologies, Grand Island, NY, USA) (Ref# A11034), horseradish peroxidase-conjugated goat anti-rabbit and anti-mouse (Santa Cruz Biotechnology, TX, USA), (sc-2005 and sc-2004)) were used. The test drug; BSB (catalog number: 14462), ROT (catalog number: R8875), glutathione (GSH) assay kit (catalog number: CS0260) and other analytical grade reagents used in this study were obtained from Sigma Aldrich, St. Louis, MO, USA.

### 2.2. Experimental Animals

To perform the experiments, five to six months old male adult albino Wistar rats weighing between 280–300 g were included in the current study. The animals were procured from the College of Medicine and Health Sciences animal research facility of the United Arab Emirates University, Al Ain, United Arab Emirates. The animals were kept at standard experimental animal housing conditions in polyacrylic cages. The animals were given food and water *ad libitum* and maintained on a 12 h light/dark cycle. The animal experimental (Ethics approval No.: ERA_6153) protocols and procedures were approved by the Animal Ethics Committee of United Arab Emirates University, United Arab Emirates.

### 2.3. Experimental Procedure

**Figure 1:**
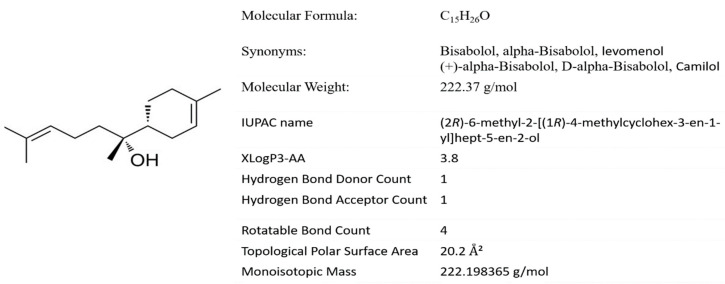
Chemical structure of α-bisabolol and physicochemical properties.

For the induction of experimental PD in rats, a well-standardized and established dosage schedule was followed wherein ROT was injected 2.5 mg/kg body weight intraperitoneally for 4 weeks. The dosage regimen of ROT including dose, duration and mode of administration employed in the present study was in line with our earlier published papers and previously other described methods [30]. The physicochemical properties indicative of druggability and chemical structure of BSB is presented in Figure 1. In brief, ROT was initially prepared as 50X stock solution in dimethyl sulfoxide and was diluted in sunflower oil (vehicle) in order to achieve the final concentration of 2.5 mg/mL. BSB was diluted in oil to prepare the dose of 50 mg/kg body weight. BSB dose in the present study was selected based on previous studies [31] and was injected intraperitoneally 30 min before ROT administration, once daily for 4 weeks. Administration of oil alone (vehicle) to the animals served as control. The four groups of animals are allocated as follows; Group I: Vehicle injected normal control group (CON), Group II: Rotenone injected group (ROT), Group III: BSB injected 30 min before to rotenone administered group (BR) and Group IV: BSB injected 30 min before to vehicle injected normal control group (BSB).

### 2.4. Tissue Collection

The rats assigned to each experimental group were sacrificed two days after the last injected dose of BSB or ROT to make sure an adequate washout of injected drugs from the systemic circulation. For euthanization of animals, an anesthetic agent; sodium pentobarbitone (40 mg/kg body weight) was injected intraperitoneally and cardiac perfusion was performed using phosphate-buffered saline (0.01 M, pH 7.4) for washing out the blood. After perfusion of the anaesthetized animals, the brain was removed as rapidly as possible, and the brain hemispheres were separated. From one hemisphere, the striatum and midbrain region were separated out on ice-chilled plate and the tissues for biochemical studies were snap-frozen in liquid nitrogen until processing. The other hemisphere was post-fixed using 4% paraformaldehyde solution for two days and subsequently added 30% sucrose solution at 4 °C until the tissues completely sunk down, before sectioning.

### 2.5. Biochemical Studies

Samples of brain tissue (midbrain) were homogenized in the potassium chloride buffer added with protease and phosphatase inhibitor (Thermo Fisher Scientific, Rockford, IL, USA, catalog number: 78420) using a handheld tissue homogenizer individually for each group of samples. Thereafter, the sample homogenate was centrifuged at 14,000 *g* for 20 min at 4 °C to obtain the post-mitochondrial supernatant that was used for biochemical estimations. The quantifications of lipid peroxidation marker; malondialdehyde (MDA), endogenous non-enzymatic antioxidants; glutathione (GSH) and enzymatic antioxidants; superoxide dismutase (SOD), and catalase (CAT) were measured by the commercially available kits. The levels of proinflammatory cytokines (IL-1β, IL-6 and TNF-α) were also measured by commercial enzyme-linked immunosorbent assay (ELISA) kits.

### 2.6. Assessment of Malondialdehyde (MDA), a Product of Lipid Peroxidation

The lipid peroxidation product was estimated by quantifying the levels of MDA assay kit acquired from North West life sciences, Vancouver, WA, USA (catalog number: NWK-MDA01). In brief, the calibrators or samples (250 μL) were incubated in the mixture of acid reagent and thiobarbituric acid (250 μL) and further strongly mixed with vortex mixer. Subsequently, samples were incubated at 60 °C for 60 min. Following incubation samples were centrifuged at 10,000 *g* for 2–3 min. The resultant reaction mixture formed was taken into a cuvette and the absorbance was read at 532 nm using VersaMax^TM^, tunable microplate reader (Molecular Devices, San Jose, CA, USA). The results derived from the absorbance were expressed as µM MDA.

### 2.7. Assessment of Glutathione (GSH) Levels

The level of GSH in sample homogenate was measured from the commercially available kit as per the manufacturer’s protocol procured from Sigma-Aldrich, St. louis, MO, USA (catalog number: CS0260). Samples were first deproteinized by 5-sulfosalicylic acid solution (5%) and centrifuged to eliminate the precipitated protein. Further supernatant was then employed to measure the level of GSH. Samples or standards (10 μL) were incubated for 5 min in 150 μL of the working mixture containing assay buffer with 5, 5′-dithiobis (2-nitrobenzoic acid) and glutathione reductase in a 96-well microplate. To each well of the microplate, a working solution; NADPH (50 μL) was added and mixed strongly. After incubation of the plate for 5 min, the absorbance was read at 412 nm using VersaMax^TM^, tunable microplate reader (Molecular Devices, San Jose, CA, USA). The results derived from the absorbance were expressed as µM GSH.

### 2.8. Assessment of Antioxidant Enzymes Activity

The activities of endogenous antioxidant enzymes; superoxide dismutase (SOD) and catalase (CAT) were measured using commercially available assay kits purchased from Cayman Chemicals Co., Ann Arbor, MI, USA (catalog number: 706002 and 707002). The SOD activity was measured by incubating the test samples or standards (10 μL) in a 96-well plate. Thereafter, xanthine oxidase (20 μL) was mixed to the each well in order to initiate the reaction, after that plate was lightly mixed by shaking, and covered the plate and incubated at room temperature for 30 min. Absorbance was noted at 450 nm by a microplate reader. The activity of CAT was measured in a 96-well microplate by adding standards or test samples (20 μL) to the assay buffer (100 μL) and methanol (30 μL). To start the reaction, hydrogen peroxide solution (20 μL) was added and samples were incubated at room temperature for 20 min. To stop the reaction, potassium hydroxide (30 μL) was added to each well and further catalase purpald (30 μL) and catalase potassium periodate (10 μL) were added to the plate and kept on a shaker for mixing for 5 min at room temperature. The absorbance was recorded at 540 nm with a VersaMax^TM^, tunable microplate reader (Molecular Devices, San Jose, CA, USA). The activities of SOD and CAT were represented as U/mL and nmol/min/mL respectively.

### 2.9. Assessment of Nitrite Levels (NO)

The levels of total NO level were measured in the midbrain tissues with commercial kit procured from R&D system, Minneapolis, MN, USA (Catalog number: R&D KGE 001). Briefly, the reaction diluent (50 μL) were added with nitrite standard or test samples (50 μL) in a 96-well plate. Thereafter, Griess reagent (50 μL) was added in each well and mixed by gentle plate shaking. The plate was incubated for 10 min at the room temperature and absorbance was recorded at 540 nm using a VersaMax^TM^, tunable microplate reader (Molecular Devices, San Jose, CA, USA). The values of nitrite were shown as µM/mg protein.

### 2.10. Assessment of Matrix Metalloproteinase-9 (MMP-9) Activity

The measurement of MMP-9 was carried in the midbrain tissues through commercially available kit purchased from R&D system Minneapolis, MN, USA (Catalog number: R&D DY8174). Briefly, 96 well ELISA plate was coated with capture antibody (100 μL) and incubated at room temperature for overnight. Subsequently, wells were washed three times with wash buffer containing 0.05% tween 20 in PBS 0.01 M (pH 7.4). Thereafter blocking buffer (1% BSA, 300 µL) was added to the plate for 1 hour and test samples or standard (100 μL) were dispensed and incubated for two hours at room temperature. The diluted detection antibody (100 μL) was added to each well and incubated for one hour. Further, the plate was washed and the substrate was added to each well and incubated for 30 min. Finally, a stop solution containing 2N H_2_SO_4_ (50 μL) was added to each well and the plate was shaken gently to ensure uniform mixing. The absorbance of the well was recorded instantly at 450 nm with a VersaMax^TM^, tunable microplate reader (Molecular Devices, San Jose, CA, USA). The values of MMP-9 were expressed as pg/mL.

### 2.11. Assessment of Proinflammatory Cytokines

The proinflammatory cytokines interleukin-1β (IL-1β), interleukin-6 (IL-6), and tumor necrosis factor-alpha (TNF-α) were estimated using commercial ELISA kits purchased from R&D system Minneapolis, MN, USA (Catalog numbers: R&D DY501, R&D DY506 and R&D DY510). In short, 96 well ELISA plate was coated with the working concentration of capture antibody (100 μL) at room temperature for overnight. Subsequently each well was properly rinsed with the wash buffer (0.05% tween 20 in PBS 0.01 M pH7.4) using automated washer and aspirator. The plate was then blocked with reagent diluent (1% bovine serum albumin in PBS) 300 μL for one hour and then washed with wash buffer. Thereafter, 100 μL of standards or samples were added to the well and incubated for two hours. Following incubation, 100 μL of detection antibody was added to the each well and then re-incubated for two hours at the room temperature. Streptavidin horseradish peroxidase antibody was diluted (100 μL in a ratio of 1:200) and added to each well and incubated at the room temperature for 20 min. Subsequently, the substrate solution (100 μL) was added to each well of the microplate and incubated for 20 min. Thereafter, a stop solution containing 2N H_2_SO_4_ (50 μL) was added to each well and the plate was lightly shaken to ensure good mixing. Using a VersaMax^TM^, tunable microplate reader (Molecular Devices, San Jose, CA, USA), the absorbance at 450 nm was recorded instantly of each well. The values of the cytokine levels were represented as pg/mL.

### 2.12. Immunocytochemistry of Tyrosine Hydroxylase (TH)

The rat brain from each experimental group was collected as mentioned earlier and sectioned at the level of stratum and SN for TH immunostaining. Coronal sections with 14 μm thickness were cut through cryostat (Leica, Wetzlar, Germany). The sections were then rinsed two times with PBS (0.01 M, pH 7.4) and for 1 h incubated with the blocking solution (10% normal goat serum in PBS, 0.3% Triton-X 100). Afterward, the sections were incubated with the primary antibody against TH (polyclonal rabbit anti-TH 1:500) at 4 °C for overnight. The sections were then rinsed and incubated with secondary antibody biotinylated anti-rabbit (1:1000) at room temperature for an hour. To visualize the TH immunoreactivity, sections were incubated with avidin-biotin complex (Vector Laboratories Ltd. Burlingame, CA, USA) and 3,3′ diaminobenzidine (DAB). In the last step, the slides were cover-slipped using DPX mounting medium and kept overnight to dry. The slides were then observed under a light microscope (Olympus, Hamburg, Germany). For the analysis of TH positive neurons and fibers, images were captured under light microscope and stored in the computer.

### 2.13. Immunofluorescence Staining of GFAP and Iba-1

The GFAP and Iba1 immunofluorescence staining were carried out in the striatum to assess the activation of astrocytes and microglia respectively. Striatum sections were rinsed two times with PBS and incubated for 1 h in blocking solution (10% normal goat serum and 0.3% Triton-X 100 in PBS). Afterwards, sections were washed thrice and polyclonal rabbit anti-GFAP (1:1000) and anti-Iba-1 (1:1000) antibodies were added and incubated for overnight at 4 °C. The sections were again washed and incubated with fluorescently labelled secondary antibody Alexa Fluor® 488 anti-rabbit for an hour at the room temperature. Subsequently, the sections were rinsed and mounted with anti-fade mounting medium Fluoroshield™. The images were captured using a fluorescence microscope, EVOS FL (Thermo Fisher Scientific, Waltham, MA, USA).

### 2.14. Assessment of Tyrosine Hydroxylase-immunoreactive (TH-ir) DA Neurons and TH-ir DA Fibers

The assessment of ROT-induced TH-ir neurons loss in the SNc region was carried out as previously reported [32]. Briefly, in the SNc area, three different levels (−4.8, −5.04, and −5.28 mm of bregma) were selected and the number of TH-ir neuron was counted and an average was presented as a percentage. The loss of TH-ir fibres was measured by quantifying the optical density of TH-ir DA fibres in the striatum (adjacent to 0.3 mm of bregma) with Image J software (NIH, Bethesda, MD, USA). Optical density of the fibres was assessed in three different, equally sized fields of each striatum section (three sections/rat). The average of these three areas were calculated and represented as a percentage in reference to the control. The measurement of the optical density of overlying cortex was considered as a background during analysis and subtracted from the value generated from the striatum. The counting of TH-ir neurons and measurement of the optical density of the TH-ir fibres were performed by evaluator masked to the experimental groups.

### 2.15. Assessment of Activated Astrocytes and Microglia

The ROT-induced activation of glial cells was assessed in the striatum. At least three striatal coronal sections at a parallel level from each rat were taken into consideration to analyze the number of activated astrocytes and microglia. Increased immunofluorescence intensity and extended glial processes were taken into account for the activation of astrocytes and microglia. Image J software was used for the quantification of activated astrocytes and microglia in three, randomly chosen, different, equally sized fields.

### 2.16. Western Blot Analysis of COX-2, iNOS, Bcl2, Bax, Cleaved Caspase-3 and 9, and Cytochrome-C

The striatal tissues from rat’s brain of each group were used to prepare the homogenate in the RIPA buffer (Merck Millipore, Burlington, MA, USA, catalog number-20188) and cytoplasmic fractions was obtained. Using mitochondrial isolation kit purchased from Abcam, Cambridge, MA, USA (catalog number-110168), and the mitochondrial fraction of the striatal tissues was prepared. The cytoplasmic or mitochondrial fractions from each striatal sample with equal amounts of protein (35 μg) were separated by gel electrophoresis employing SDS-polyacrylamide (10–12%). Then after, the proteins were transferred onto the PVDF membrane and incubated with specific primary antibodies polyclonal rabbit anti-iNOS (1:1000), anti-COX-2 (1:1000), anti-Bax (1:1000), anti-Bcl-2 (1:500), anti-cleaved caspase-3 (1:500), anti-cleaved caspase-9 (1:500), anti-cytochrome-C (1:1000), anti-VDAC (1:2000) and anti-β-actin (1:2000) and kept overnight at 4 °C on shaker for mixing. The PVDF membranes were washed and incubated for 1 h at room temperature with their corresponding secondary antibodies (anti-rabbit or anti-mouse IgG) (sc-2005 and sc-2004) conjugated to horseradish peroxidase. Using an enhanced chemiluminescence pico kit (Thermo Fisher Scientific, Rockford, IL, USA, catalog number: 34580), the protein bands developed were visualized and their intensity was determined by densitometry and quantified with Image J software.

### 2.17. Estimation of MC-I and Adenosine Triphosphate (ATP)

The complex I activity in the striatum was determined by the method of Spinazzi et al. [33] with slight modifications. Briefly, 100 µL of mitochondrial fraction was added to the reaction mixture consists of 100 µL potassium phosphate buffer (0.5 M, pH 7.5), 60 µL of fatty acid-free BSA (50 mg/mL), 30 µL of sodium azide (10 mM) and 10 µL of NADH (10 mM). The volume is adjusted to 994 µL with distilled water. After gentle mixing, the reaction was started by adding 6 µL of ubiquinone_1_ (10 mM). The decrease in absorbance was read at 340 nm for 4 min. MC-I activity was expressed as nM of NADH oxidized/min/mg protein. ATP concentration in the mitochondrial fraction was measured fluorimetrically by the method of Williams and Coorkey [34]. The incubation mixture contained 2 mL of triethanolamine buffer (50 mM, pH 7.4), 10 mM magnesium chloride, 5 mM ethylene diamine tetra acetic acid, 10 μL of nicotinamide adenosine diphosphate (NADP) (10 mM) and 5 μL of glucose-6-phosphate dehydrogenase (0.2 mg/mL). It was mixed thoroughly and the fluorescence was recorded. Then 5 μL of hexokinase (2 mg/mL) and 10 μL of adenosine triphosphate (ATP) (1 mM) were added and the increase in fluorescence was recorded at 340 nm using multimode plate reader, Infinite® 200PRO (TECAN Ltd., Männedorf, Switzerland). The ATP content was expressed as nM/mg protein.

### 2.18. Estimation of Mitochondrial Lipid Peroxidation

The concentration of thiobarbituric acid reactive substances (TBARS) in the mitochondrial fraction was estimated by the method of Fraga et al. [35]. 100 µL of the mitochondrial fraction was treated with 200 µL of thiobarbituric acid (TBA)–trichloro aectic acid (TCA)–hydrochloric acid (Hcl) reagent and mixed well. The mixture was kept in a boiling water bath for 15 min. After cooling, the tubes were centrifuged for 10 min and the supernatant was read at 535 nm against the reagent blank using VersaMax^TM^, tunable microplate reader (Molecular Devices, San Jose, CA, USA). The concentration of lipid hydroperoxides (LOOH) in the mitochondrial fraction was estimated by a previously described method [36]. To 0.2 mL of mitochondrial fraction, 1.8 mL of FOX reagent was added and incubated for 30 min at room temperature. The developed color was read at 560 nm using VersaMax^TM^, tunable microplate reader (Molecular Devices, San Jose, CA, USA).

### 2.19. Estimation of Protein Concentration

In each sample, the protein content was determined using Pierce™ BCA protein assay kit (Thermo Fisher Scientific, Rockford, IL, USA, catalog number-23225) following the protocol of manufacturer’s kit.

### 2.20. Total Antioxidant Activity (2, 2-azinobis-(3-ethyl-benzothiazoline-6-sulfonic acid) (ABTS) of BSB

Total antioxidant potential of BSB and the control caffeic acid (CA) were determined by scavenging ABTS^+^ as described by Miller et al. [37]. The reaction mixture contained ABTS^+^, BSB and CA (20–100 μM) in phosphate buffer. The absorbance was measured at 734 nm using Ultrospec 7000 spectrophotometer (Biochrom US, Holliston, MA, USA).

### 2.21. Statistical Analysis

The values were expressed as the mean ± SEM. One-way analysis of variance (ANOVA), followed by Tukey’s test or Duncan’s Multiple Range Test (DMRT) was used to analyze the data of all parameters, unless otherwise mentioned. GraphPad InStat software (GraphPad InStat, La Jolla, CA, USA) was used to analyze and calculate the statistical significance between various groups. The criteria for statistical significance were set at *p* < 0.05 for all the statistical tests.

## 3. Results

### 3.1. BSB Mitigates the Loss of TH-ir Neurons in the SNc and TH-ir Fibres in the Striatum

**Figure 2:**
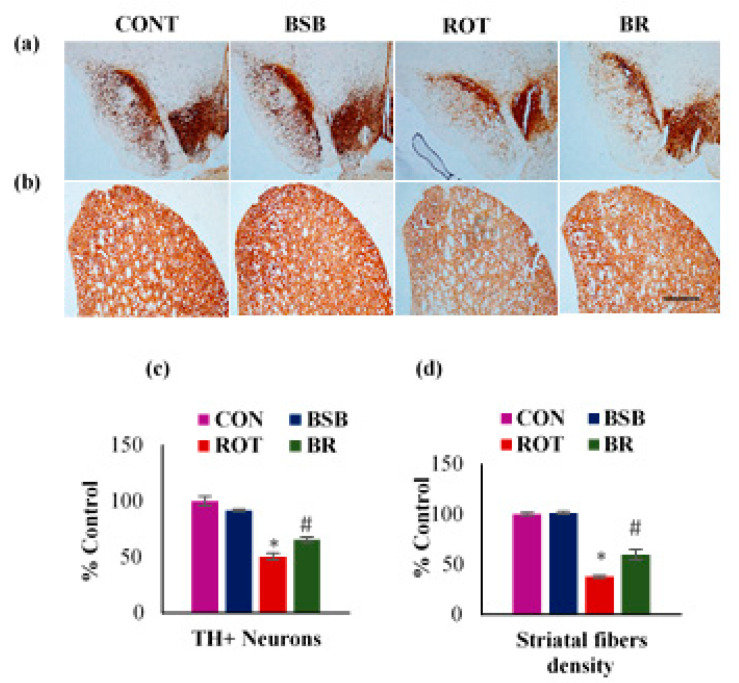
The number of TH-ir neurons and density of TH-ir fibres were determined in the substantia nigra (SNc) and striatum respectively (**a**,**b**). There was a significant decreased number of TH-ir neurons are found in the ROT injected rats when compared to control rats (CON). However, BSB treatment to ROT injected rats showed a significant increased number of TH-ir neurons compared to ROT alone injected rats (a). Similarly, a significant decrease in the TH-ir striatal fibres expression was observed in ROT injected rats when compared to control rats. Moreover, BSB treatment to ROT injected rats showed enhanced expression of TH-ir fibres in the striatum (**b**). The scale bar is 100 µm. The quantitative representation of the result of TH-ir neurons in SNc and measurement of TH-ir fibres density are shown (**c**,**d**). Each group contained three rats and the data were expressed as percent mean ± SEM. * *p* < 0.05 CON vs ROT; # *p* < 0.05 ROT vs. BR (One-way ANOVA followed by Tukey’s test).

To evaluate the neuroprotective effects of BSB on ROT-induced loss of the nigrostriatal system in rats, immunocytochemistry of TH was carried out to assess the TH-ir dopaminergic neurons in SNc and TH-ir dopaminergic fibres in the striatum (Figure 2a,b). ROT injected rats significantly (*p* < 0.05) reduced the decrease of dopaminergic neurons in the SNc when compared to ROT injected rats. However, BSB treatment to ROT injected rats significantly (*p* < 0.05) reduced the decrease of dopaminergic neurons in the SNc when compared to ROT injected rats (Figure 2c). The dopaminergic neurons have their nerve terminals spread to the striatum and these terminals possesses dopamine transporters [38]. Dopaminergic degeneration in SNc leads to a decrease in dopamine transporter in the striatum that eventually reconfirm the loss of neurons. Further, we assessed the density of dopaminergic nerve terminals fibres (TH-ir) in the striatum. The results showed significant (*p* < 0.05) in the density of TH-ir fibres in striatum of ROT injected rats when compared to vehicle treated control rats. Whereas, BSB treatment to ROT injected rats exhibited significant (*p* < 0.05) rise in the density of the striatal fibres when compared to ROT injected rats (Figure 2d). Moreover, rats treated with BSB alone showed no effect on the TH-ir neurons and fibres as compared to vehicle-treated control rats in the SNc and striatum respectively.

### 3.2. BSB Attenuates Lipid Peroxidation and Improved Levels and Activities of Antioxidants in the Midbrain

**Figure 3:**
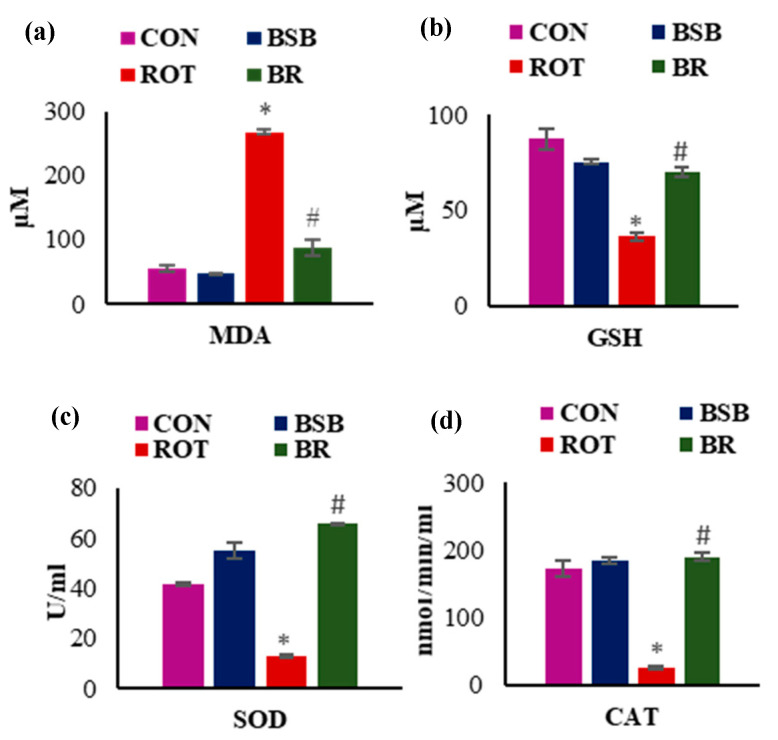
Change in the levels of MDA (**a**), GSH (**b**), SOD (**c**) and CAT (**d**) in the mid brain following ROT administration. ROT injected rats showed a significant increase in the MDA level and reduction in the GSH level compared to the vehicle treated control (CON) rats. Treatment with BSB to the ROT injected rats significantly decreased MDA, and increased GSH content. Furthermore, ROT injections also cause significant reduction in the activity of SOD and CAT when compared to the vehicle treated control rats. BSB treatment to ROT injected rats significantly increased the activity of SOD and CAT as compared to the ROT injected rats. The values are expressed as the mean ± SEM (n = 6–8). * *p* < 0.05 CON vs ROT; # *p* < 0.05 ROT vs. BR (One-way ANOVA followed by Tukey’s test).

Further, the level of MDA, an indicator of lipid peroxidation and GSH level, a primary antioxidant substrate of glutathione redox cycle was measured. Injections of ROT caused significant (*p* < 0.05) rise in MDA levels in the midbrain when compared to vehicle-injected control rats. Whereas, BSB treatment to ROT injected rats significantly (*p* < 0.05) reduced MDA levels when compared to ROT injected rats (Figure 3a). As shown in Figure 3b, the level of GSH was significantly (*p* < 0.05) reduced in ROT injected rats compared to vehicle-injected control rats. Whereas, BSB treatment to ROT injected rats showed significantly (*p* < 0.05) increased levels of GSH as compared to ROT injected rats. Animals treated with BSB alone did not show significant changes in the levels of MDA and GSH when compared to vehicle-injected control animals. To provide the further evidence in favor of antioxidant efficacy of BSB, we evaluated the activity of antioxidant enzymes SOD and CAT. The results showed that ROT injection significantly (*p* < 0.05) decreased the activities of SOD (Figure 3c) and CAT (Figure 3d) when compared to vehicle-injected control rats. Moreover, BSB treatment to ROT injected animals significantly (*p* < 0.05) increased the SOD (Figure 3c) and CAT (Figure 3d) activities. Whereas, rats treated with BSB alone did not cause significant changes in the activities of SOD and CAT in comparison with control animals. These oxidative stress related parameters were carried out to determine whether the protective effects of BSB against ROT-induced loss of the dopaminergic neurons are due to its potent antioxidant properties. An elevated level of MDA in the midbrain indicates occurrence of lipid peroxidation, an important pathogenic event of onset of oxidative stress that correlates with the reduction of tripeptide antioxidant GSH. Moreover, BSB treatment profoundly reverse the diminished activities of antioxidant enzymes caused by ROT. Collectively, this data endorses the antioxidant property of BSB that possibly plays a key role in the neuroprotective effects against ROT.

### 3.3. BSB Inhibits the Levels of Total Nitric Oxide (NO) and MMP-9

**Figure 4:**
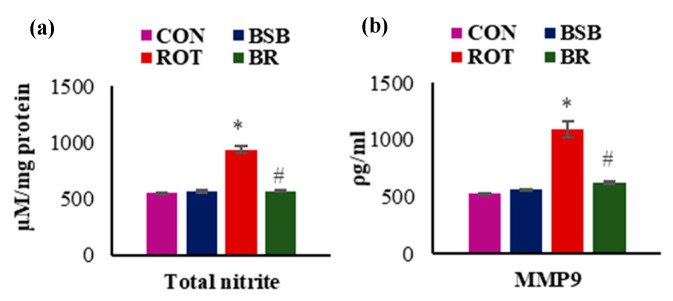
Change in the levels of total NO (**a**) and MMP-9 (**b**) in the mid brain following ROT administration. The levels of NO and MMP-9 was significantly increased in the ROT injected rats compared to control rats. Moreover, rats injected with ROT and treated with BSB showed significantly decreased levels of NO and MMP-9. The values are expressed as the mean ± SEM (n = 7–8). * *p* < 0.05 CON vs ROT; # *p* < 0.05 ROT vs. BR (One-way ANOVA followed by Tukey’s test).

In addition to antioxidant enzymes, we also estimated total NO and MMP-9 levels in the midbrain tissues. The results showed that ROT injections caused significant (*p* < 0.05) increase in the levels of total NO (Figure 4a) and MMP-9 (Figure 4b). Whereas, BSB treatment to ROT injected animals significantly (*p* < 0.05) decreased NO (Figure 4a) and MMP-9 levels (Figure 4b) when compared to ROT injected animals. Moreover, BSB alone treated animals did not show significant changes in the levels of NO and MMP-9 in comparison with control animals.

### 3.4. BSB Inhibits the Activation of Glial Cells and Attenuates the Release of Proinflammatory Cytokines in the Striatum

**Figure 5:**
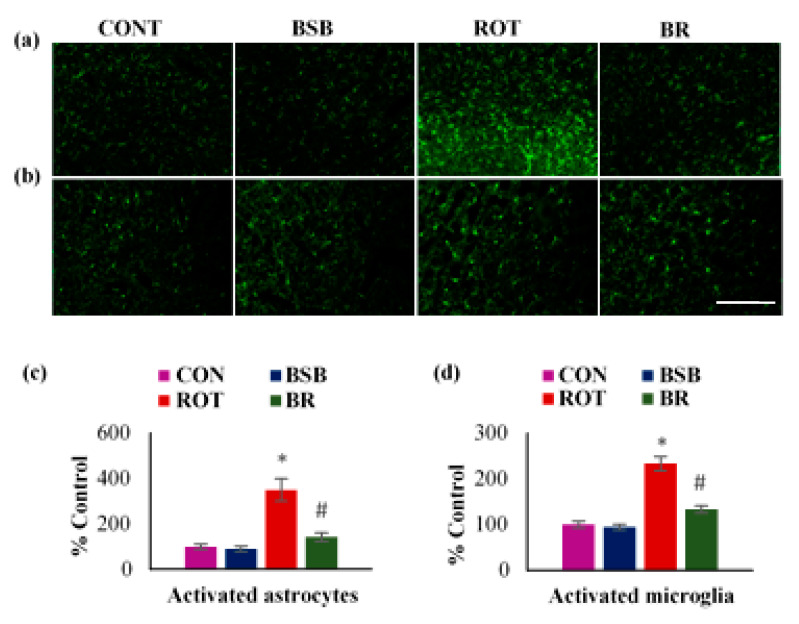
The immunofluorescence expression of GFAP and Iba-1 in striatum was determined. A profound expression of GFAP-positive astrocytes (**a**), and Iba-1-positive microglia (**b**), was observed in the ROT-injected rats in comparison with vehicle treated control (CON) rats. Interestingly, BSB treatment to ROT injected rats showed decreased expression of GFAP and Iba-1 compared to ROT injected rats (scale bar = 200 µm). The quantitative representation of the result of activated astrocytes and microglia in the striatum is shown (**c**,**d**). Each group contained three rats and the data were expressed as percent mean ± SEM. * *p* < 0.05 CON vs ROT; # *p* < 0.05 ROT vs. BR (One-way ANOVA followed by Tukey’s test).

The activation of glial cells is accounted for the induction of inflammation in the brain. Following ROT injection, activation of glial cells was observed and it indicates the inflammation response in the striatum (Figure 5a,b). The immunofluorescence staining and subsequent quantification of GFAP (a marker for astrocytes) and Iba1 (a marker for microglia) showed a significant (*p* < 0.05) increment in number of activated astrocytes and microglia in the ROT injected animals as compared to vehicle-injected control rats (Figure 5c,d). Whereas, ROT injected animals treated with BSB displayed significant (*p* < 0.05) decline in number of activated astrocytes and microglia. There was no remarkable effect on astrocytes and microglia in the animals treated with BSB alone.

**Figure 6:**
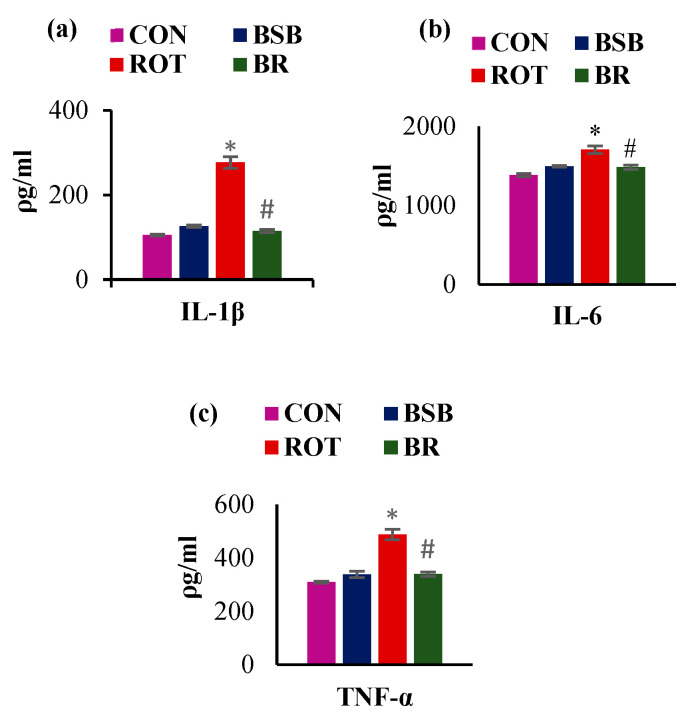
Concentration of proinflammatory cytokines IL-1β (**a**), IL-6 (**b**), and TNF-α (**c**) in the mid brain of rats injected with ROT. There was a significant increase in the concentration of IL-1β, IL-6 and TNF-α in ROT treated rats compared with control. However, BSB treatment to ROT injected rats significantly decreased these proinflammatory cytokines compared to ROT injected rats. There was no significant difference observed in the concentration of IL-1β, IL-6 and TNF-α of BSB treated rats compared to control. The values are expressed as mean ± SEM (n = 6–7). * *p* < 0.05 CON vs. ROT; # *p* < 0.05 ROT vs. BR (One-way ANOVA followed by Tukey’s test).

Sustained activation of glial cells following systemic chronic injection of ROT leads to increased secretion of proinflammatory cytokines including IL-1β, IL-6, and TNF-α which further amplify the cascade of neuroinflammation [39]. Therefore, we quantified the levels of these cytokines in the midbrain of ROT injected rats. A significant (*p* < 0.05) rise in the levels of IL-1β, IL-6, and TNF-α was observed in the ROT injected rats as compared to vehicle-injected control rats. Whereas, BSB treatment to ROT injected rats significantly decreased (*p* < 0.05) the enhanced levels of these pro-inflammatory cytokines, IL-1β (Figure 6a), IL-6 (Figure 6b), and TNF-α (Figure 6c). Moreover, BSB alone group rats did not show remarkable effect on the release of these studied proinflammatory cytokines.

### 3.5. BSB Attenuated the ROT-induced Expression of Inflammatory Mediators COX-2 and iNOS in the Striatum

**Figure 7:**
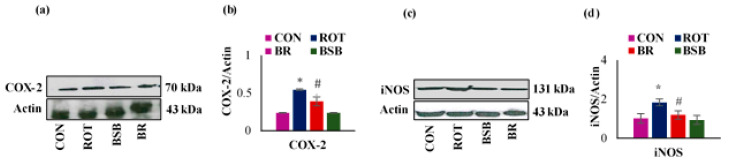
The protein expression and the densitometric analysis of COX-2 and iNOS in the striatal tissues (**a**–**d**). The quantification of the band intensity showed that a significant increase in the expression level of COX-2 and iNOS in the ROT administered rats when compared as vehicle-injected control rats (CON). However, BSB treatment to ROT injected rats displayed a significant decreased expression of COX-2 and iNOS compared to ROT injected rats. Whereas, rats treated with BSB alone did not shown marked difference in the expression level of COX-2 and iNOS. The values are expressed as the mean ± SEM (n = 3). * *p* < 0.05 CON vs. ROT; # *p* < 0.05 ROT vs. BR (One-way ANOVA followed by DMRT).

Further, we studied the protein expressions of inflammatory enzymes mediators such as COX-2 and iNOS in tissue lysates prepared from the striatum and the densitometric analysis (Figure 7a–d). A significant (*p* < 0.05) rise in the expression of COX-2 was observed in ROT injected rats when compared to the vehicle-injected control rats. However, BSB treatment to ROT injected rats displayed significant reduction in the expression of COX-2 as compared to ROT injected rats (Figure 7a,b). Similarly, ROT injected rats also exhibited a significant (*p* < 0.05) increase in the level of iNOS when compared to the vehicle-injected control rats. Interestingly, BSB treatment to ROT injected rats significantly decreased (*p* < 0.05) the level of iNOS compared to ROT-injected rats (Figure 7c,d). BSB alone treated rats did not show remarkable changes in the expression of COX-2 and iNOS when compared to control rats.

### 3.6. BSB Attenuates ROT-Induced Expression of Apoptotic Markers Bax, Bcl-2, Cleaved Caspase-3 and 9

**Figure 8:**
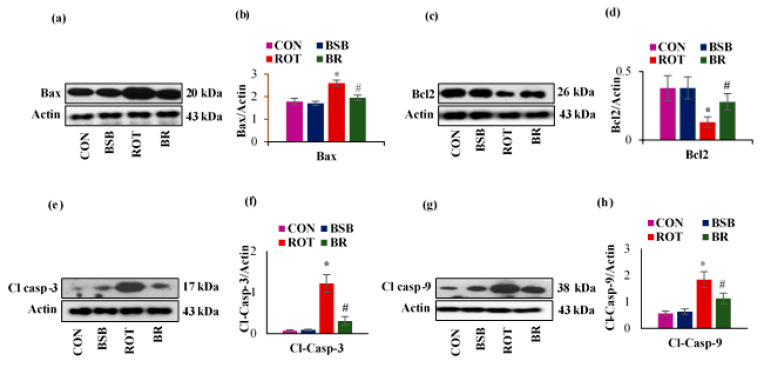
The protein expression of anti-apoptotic marker Bcl-2 and proapoptotic markers such as Bax, cleaved caspase-3 and 9 was estimated in the striatal tissues (**a**–**h**). The quantification of the blots showed that significant decrease in Bcl-2 expression (**c**,**d**) and significant increase in Bax (**a**,**b**), cleaved caspase-3 (**e**,**f**) and cleaved caspase-9 expression (**g**,**h**) in the ROT injected rats when compared to control (CON) rats. Whereas, BSB treatment significantly corrected the expression of these antiapoptotic and proapoptotic proteins in the rats, which were challenged with, ROT. Moreover, BSB alone treatment to rats had no marked effects on the expression levels of these apoptotic markers. Values are expressed as the mean ± SEM (n = 3). * *p* < 0.05 CON vs. ROT; # *p* < 0.05 ROT vs BR (One-way ANOVA followed by DMRT).

Chronic ROT administration has been shown to induce the apoptotic cell death in rats [40]. We also examined the ROT-induced expression of anti-apoptotic protein Bcl-2 and pro-apoptotic proteins Bax cleaved caspase-3 and 9 in the striatal tissues and the densitometric analysis of the developed band (Figure 8a–h). In ROT-injected rats, a significant (*p* < 0.05) decline in the expression level of Bcl-2 was observed as compared to vehicle-injected control rats. Interestingly, BSB treatment to ROT injected animals exhibited a significant (*p* < 0.05) increase in the expression level of Bcl-2 when compared to ROT injected rats (Figure 8b). In contrast, in ROT injected rats a significant (*p* < 0.05) increase in the proapoptotic proteins viz. Bax, cleaved caspase-3 and 9 was observed when compared to control rats which is in agreement with previous published report by Chiu et al. [41]. Whereas, BSB treatment to ROT injected rats displayed significant (*p* < 0.05) reduction in the expression of these proapoptotic proteins compared to ROT injected rats (Figure 8c–e). BSB alone injected rats had no significant effects on the expression of proapoptotic and antiapoptotic proteins compared to control rats.

### 3.7. BSB Attenuates ROT-Induced Mitochondrial Dysfunction

**Figure 9:**
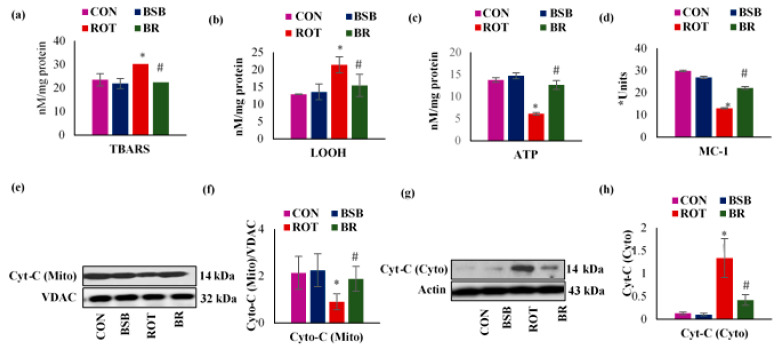
Concentrations of TBARS (**a**) and LOOH (**b**) in the mitochondrial fraction of striatum. Levels of ATP in the mitochondrial fraction of striatum (**c**) and effect of BSB on MC-I activity in the mitochondrial fraction of striatum (**d**). ROT-injected rats showed a significant decrease in the activity of MC-1 when compared to control rats (CON). In contrast, BSB treatment to ROT injected rats significantly restored the activity of MC-I (*Units-nM of NADH oxidized/min/mg protein) (**d**). Mitochondrial and cytosolic protein expression of cytochrome-C in the striatum and the densitometric analysis (**e**–**h**). ROT-injected rats showed a significant decrease in the expression of mitochondrial cytochrome-C with significant increase in the expressions of cytosolic cytochrome-C compared to control rats (CON). In contrast, BSB treatment to ROT injected rats significantly restored the expression of cytochrome-C compared to ROT alone injected rats. The values are expressed as mean ± SEM (n = 3). * *p* < 0.05 CON vs. ROT; # *p* < 0.05 ROT vs. BR (One-way ANOVA followed by DMRT).

ROT administration showed a significant (*p* < 0.05) increase in the concentrations of mitochondrial TBARS and LOOH with declined mitochondrial ATP level in the striatum (Figure 9a–c). Also, it is widely known that ROT is MC-I inhibitor [42], so we attempted to analyze the activity of mitochondrial MC-I in the mitochondrial fraction of the striatum. The activity of MC-I was significantly decreased (*p* < 0.05) in ROT injected rats as compared to control group rats (Figure 9d). Furthermore, ROT triggers the release of cytochrome-C from striatal mitochondria into the cytosol as evidenced by decreased protein expressions of cytochrome-C in the mitochondrial fraction with increased protein expressions of cytochrome-C in the cytosolic fractions. Whereas, BSB treatment inhibits cytochrome-C release compared to normal control rats (Figure 9e–h). Moreover, BSB alone treated rats did not shown remarkable changes in the activity of MC-I and cytochrome-C when compared to control rats.

### 3.8. The In Vitro Antioxidant Potential of BSB

**Figure 10:**
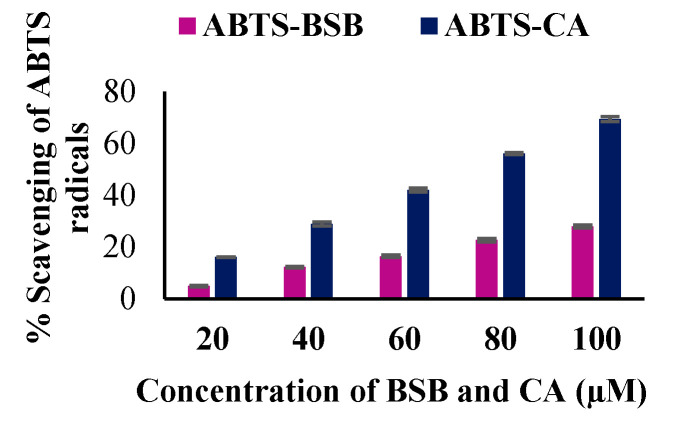
The in vitro scavenging effects of BSB and CA on ABTS radicals (Total antioxidant activity). Each column is the average of triplicate experiments.

We have performed in vitro ABTS assay to detect the antioxidant potential of BSB. Caffeic acid, a well-known dietary phenol and a potent antioxidant was used as control. BSB scavenges ABTS radical in vitro in a concentration dependent manner (20, 40, 60, 80 and 100 µM). The percentage scavenging effect of BSB at various concentrations 20, 40, 60, 80 and 100 µM were observed to be 4.95, 12.2, 16.7, 22.69 and 27.89 respectively. Also, the percentage scavenging effect of CA at various concentrations 20, 40, 60, 80 and 100 µM were observed to be 16.07, 28.89, 42.01, 56.08 and 69.38) respectively (Figure 10).

## 4. Discussion

The results of the current study demonstrate the neuroprotective effects of BSB in rat model of ROT-induced PD ascribable to its potent antioxidant, anti-inflammatory and anti-apoptotic properties. BSB is a naturally occurring monocyclic sesquiterpene alcohol component in the essential oil of several plants of the Asteraceae family. BSB is commonly used in foods, beverages, cosmetics and dermatological preparations for human use [43]. BSB also exhibit many pharmacological activities, such as antitumor activity [44], blockade of Ca^2+^ [45], antinociceptive and anti-inflammatory activities [17], antioxidant activity [17], wound-healing properties [46], and inhibitory actions on 7-nicotinic acetylcholine receptors [47]. It appears that BSB has multiple pharmacological properties and exert protective effects against varied chemical and biological insults. Thus, BSB could be a potential neuroprotective agent against PD, along with its many other functions.

Since PD is one of the chronic progressive neurodegenerative disease and the symptoms become worse over the time, therefore the efficacy of long-term administration of BSB was studied in a chronic rat model of ROT-induced PD, which reasonably recapitulates the advanced pathological and phenotypic symptoms of PD during four weeks of its administration [48]. ROT, an inhibitor of MC-I was used in developing animal model of PD for the first time in the 1980s and continue to use in pharmacological studies for drug discovery and elucidation of mechanisms [49].

In this study, co-treatment of BSB (50 mg/kg, i.p.) successfully attenuated ROT-induced nigrostriatal degeneration. ROT is well known to produce these features [50] by inducing loss of the dopaminergic neurons in SNc, thereby diminishing the striatal dopaminergic input. In this context, the present study results reveal a significant reduction of TH-ir neurons in the SNc and TH-ir fibers in the striatum of ROT injected rats and this reduction was significantly ameliorated by co-treatment with BSB.

In addition, ROT exerts its effects by inhibiting the MC-I that eventually leads to the generation of ROS including superoxide, hydroxyl radical and peroxynitrite. Excessive generation of ROS is critical in the SNc because of low antioxidant defenses relating to a very high level of dopamine metabolism [48]. The present study reveals a significant decline in the activity of MC-I in the striatum of ROT injected rats. Elevated ROS production triggers mitochondrial lipid peroxidation and altered membrane potential as confirmed by increased mitochondrial TBARS and LOOH in the striatum along with the release of mitochondrial cytochrome-C into the cytosol thereby inducing mitochondrial pathway of apoptosis [51]. Declined mitochondrial respiration, especially impaired ATP production due to altered MC-I activity has been involved in the pathology of PD which leads to the production of ROS resulting in cellular dysfunction and death [52]. Interestingly, BSB treatment showed significantly decreased levels of mitochondrial lipid peroxidation products, increased ATP levels and reinstates the activity of MC-I. Thus, BSB modulated the MC-I activity, which is the prime target to initiate the oxidative stress mediated mitochondrial dysfunction in the brain of ROT injected animals.

We measured the oxidative/nitrosative stress parameters in the mid brain tissues such as activities of antioxidant enzymes e.g., SOD and CAT; and levels of GSH, total nitrite, and MDA in order to determine antioxidant tissue defense. The role of astrocytes, second most abundant cells in the brain after neurons against oxidative damage is exerted through releasing endogenous antioxidant species such as GSH and SOD or removing glutamate. Lipid peroxidation in tissues is a key pathogenic occurrence, arising from imbalance between generation of ROS and the accessibility of endogenous antioxidant defense to counter excessive production of ROS. We found that rats injected ROT showed a significant increase in the levels of MDA, with a concomitant reduction in the GSH in the midbrain tissues. However, BSB treatment to ROT injected rats decreased the lipid peroxidation as demonstrated by the less MDA formation followed by normalization of the GSH levels. The decrease in MDA level and concomitant return to normal level of GSH content by BSB are in agreement with an earlier published report, which has shown that BSB inhibited the lipid peroxidation and improved the non-enzymatic parameters in isoproterenol-induced myocardial infarction [53].

Further, the cells are equipped with the enzymatic defense system includes SOD and CAT, as an important element in minimizing the levels of ROS. The current study represents that ROT-induced a significant reduction in the activities of enzymes; SOD and CAT as compared to control rats. Interestingly, BSB treatment to rats challenged with ROT exhibited a significant increase in activities of SOD and CAT. Moreover, we also noticed a significant increase in total nitrite level in rats challenged with ROT, ascribed to nitric oxide generation that produces peroxynitrite, which reacts to superoxide anion leading to severe neurotoxicity [54]. BSB treatment to ROT-administered rats significantly attenuated the increased levels of nitrite. These beneficial effects of BSB against oxidative stress are ascribed to its strong antioxidant activity. Scavenging ABTS radical is one of the widely used spectrophotometric assay to detect the antioxidant potential of pure substances [55]. In our study, BSB scavenges ABTS radical in a concentration dependent manner. The highest scavenging effect of BSB at 100 µM was found to be 27.89%. Thus, BSB shows convincing antioxidant potential, which may counter the free radicals and attenuates the pathological alterations induced by ROT in rats.

To our knowledge, the current study is first in reporting that BSB decreases oxidative stress in ROT-induced dopaminergic neurodegeneration by curbing the inhibition of MC-I. Recently apoptosis has been shown to play an important mechanism of cell death in PD. This is mainly ascribed to the key markers including Bax/Bcl2 ratio and activation of caspase-3 and -9 [56]. In the postmortem brain of PD patients, alteration in apoptosis-related markers are observed in the SNc and related dopaminergic regions [57] as well as enhanced immunoreactivity of the pro-apoptotic protein, Bax [58]. Therefore, it convincingly appears that apoptosis has a central role in PD pathophysiology, thus, targeting of these molecular pathways triggered during apoptosis may lead to novel therapeutic approaches for PD [59].

Results of the present study showed significant alteration in the expression of Bax/Bcl2, in addition to increased expression of caspase-3 and caspase-9 in the striatum of ROT-injected rats compared to control rats. ROT has been shown to enhance the expression of apoptotic markers, such as Bax/Bcl-2 as well as caspase-3 and caspase-9 [60]. The sustained activation of Bax facilitates permeabilization of outer-membrane of the mitochondria, which provokes death-inducing factors resulting in both mode of cell death, apoptotic and nonapoptotic [61]. The data of the present study demonstrates that BSB attenuated ROT-induced apoptosis by augmenting the Bax/Bcl2 protein expression levels and lessening the expression of cleaved caspase-3 and 9 in the striatum of ROT injected rats. These findings are reinforced by earlier studies, which demonstrate the anti-apoptotic effect of BSB in Aβ_25-35_ induced neurotoxicity in PC12 cells [23]. Moreover, a recent study from our laboratory also reported that BSB abrogates the isoproterenol-induced myocardial infarction by attenuating the intrinsic pathway of apoptotic cell death in rats [62].

Multiple findings reported that matrix metalloproteinases (MMPs) play key roles in oxidative stress and inflammation-induced neurodegeneration. In addition to its normal physiological roles, the pathological roles of MMPs are also initiated and instituted by oxidative stress [63]. MMP-9 has been showed elevated in toxicant-induced experimental models of PD [64]. MMP-9 is particularly located in neurons and contributes to the activation of glial cells, which causes an aggressive release of proinflammatory cytokines and eventually leads to neurodegeneration in both the monkey and mouse models of PD [65]. Furthermore, MMP-9 knockout animals showed that MPTP, a neurotoxin used to induce PD resulted in reduced microglial activation and increased rate of survival of dopaminergic neurons when compared to wild type animals [65]. In light of these findings, in the present study we also observed significantly high levels of MMP-9 in the midbrain of ROT injected rats. Whereas, BSB treatment to ROT injected rats caused a significant reduction in the level of MMP-9 that is partly attributed to antioxidant property of BSB.

ROT-injected animals also displayed the occurrence of neuroinflammation, which is initiated and further sustained by numerous mechanisms including dysfunction of mitochondria and resultant formation and release of ROS to elicit the activation of glial cells [66]. Oxidative modifications of α-syn and its successive aggregation also provide a robust impetus for initiating and boosting neuroinflammation [67]. In agreement to this, a rise in the number of activated astrocytes and microglia with a parallel development in the occurrence of the pro-inflammatory cytokines such as IL-1 β, IL-6, and TNF-α in the ROT injected rats that was ameliorated following co-treatment of BSB.

COX-2 is one of the important mediator of inflammation and rise in its activity/levels have been showed to escalate significantly in the PD-affected brain [68]. Multiple studies support the hypothesis that COX-2 potentiates the cytotoxic effects not only through the ROS generated during conversion of prostaglandins-G to prostaglandins-H but also by producing pro-inflammatory prostaglandins that leads microglial activation [29]. Besides COX-2, iNOS, another enzyme participate in dopaminergic neurodegeneration by production of iNOS involving activated microglia which results in the increased formation of NO and subsequently deleterious effect on DNA and proteins [68]. The present study reveals an inhibitory effect of BSB on the ROT-induced increased expression of COX-2 and iNOS as well as NO level. Moreover, BSB was previously reported a potent inhibitor of COX-2 and iNOS in lipopolysaccharides-induced inflammation as well as in the experimental model of cerebral ischemia [69]. The present observations are supported by earlier studies, wherein ROT has been reported to increase COX-2 and iNOS expression/activity in rats [30].

## 5. Conclusions

In conclusion, the present study reveals that BSB exert potent neuroprotective effects mediating antioxidant, anti-inflammatory, antiapoptotic activities in the ROT-induced rat model of PD. The major underlying mechanism attributed to the neuroprotective effects are the restoration of MC-I that consequently results in alleviation of oxidative stress, neuroinflammation, glial cells activation, pro-inflammatory cytokine and mitigating nigrostriatal degeneration. The present study findings suggest that BSB, a monocyclic dietary sesquiterpene may be an attractive candidate of natural origin for PD to improve conventional therapies as well as provide novel disease-modifying therapeutic agents.

## Figures and Tables

**Table 1:** Primary antibodies used, source, and dilution.

*Antibody*	Host	Source/Catalogue No.	Dilution
Tyrosine hydroxylase (TH)	Rabbit	Millipore, MA, USA (AB-152)	1:500
Glial fibrillary acidic protein (GFAP)	Rabbit	Abcam, MA, USA (SAB2107063)	1:1000
Ionized calcium binding adaptor molecule 1 (Iba1)	Rabbit	Wako Chemicals, VA, USA (019-19741)	1:1000
Cytochrome-C	Mouse	Abcam, MA, USA (AB13575)	1:1000
Cyclooxygenase-2 (COX-2)	Rabbit	Abcam, MA, USA (AB52237)	1:1000
Inducible nitric oxide synthase (iNOS)	Rabbit	Sigma, MO, USA (SAB4502011)	1:1000
B-cell lymphoma *2* (Bcl-2)	Rabbit	Abcam, MA, USA (AB196495)	1:500
BCL2 Associated X, Apoptosis Regulator (Bax)	Rabbit	Santacruz, Dallas, USA (SC-526)	1:1000
Cleaved caspase-3	Rabbit	Abcam, MA, USA (AB49822)	1:500
Cleaved caspase-9	Rabbit	Cell signaling Technology, USA (9507S)	1:500
Voltage-dependent anion channel (VDAC)	Rabbit	Cell signaling Technology, USA (4661S)	1:2000
β-actin	Mouse	Millipore, MA, USA (MAB1501R)	1:2000
